# Relationships between informal caregiving, health and work in the Health and Employment After Fifty study, England 

**DOI:** 10.1093/eurpub/ckaa078

**Published:** 2020-06-03

**Authors:** E Clare Harris, Stefania D’Angelo, Holly E Syddall, Cathy Linaker, Cyrus Cooper, Karen Walker-Bone

**Affiliations:** c1 MRC Lifecourse Epidemiology Unit, University of Southampton, Southampton, UK; c2 MRC Versus Arthritis Centre for Musculoskeletal Health and Work, University of Southampton, Southampton, UK; c3 Nuffield Department of Orthopaedics, Rheumatology and Musculoskeletal Sciences, University of Oxford, Oxford, UK

## Abstract

**Background:**

To investigate the prevalence of caregiving and its relationship with work, health and socio-economic circumstances in the Health and Employment After Fifty (HEAF) study.

**Methods:**

The HEAF study comprises 8134 men and women aged 50–64 years recruited from 24 general practices. Socio-demographic, lifestyle and health characteristics and hours per week giving personal care were elicited by postal questionnaire. Objective clinical information about diagnoses/medications was retrieved from health records. Work-related and health risk factors for intense caring responsibilities (≥20 h/week vs. no hours) were explored using logistic regression with adjustment for age and social class.

**Results:**

In all, 644 (17%) men and 1153 (26%) women reported caring responsibilities, of whom 93 and 199 were intense caregivers, who were more likely to be socio-economically disadvantaged; less likely to be working and, if combining caring with working (41 men and 90 women), more likely to be part-time/working shifts, than non-carers. Men caring ≥20 h/week were more likely to have COPD and to report musculoskeletal pain, poor/fair self-rated health, depression and sleep problems. Among working women, caring ≥20 h/week was associated with these same health outcomes and also with a doctor-diagnosed mental health problem or musculoskeletal pain in the previous year.

**Conclusions:**

Caregiving is common and unequal in the HEAF cohort, with more high-intensity informal care provided by those with greater levels of socio-economic deprivation, which could affect their employment and health. Caregivers need support to lead long, healthy lives, rather than becoming care needers themselves. Employers and governments need to take caregiving into account and support it actively.

## Introduction 

Life expectancy in Europe has been increasing and, combined with reducing birth rates, has changed the shape of our societies with growing proportions of older people relative to those of working age.^1^ In response, governments have legislated to encourage people to work to older ages. With an ageing population also comes an increased demand for informal care.^2^ Informal caregivers are usually unpaid, often a relative and are providing support for activities of daily living for an individual with a severe disability, chronic illness or dementia. According to one estimate, one in three Europeans provide some informal care and 8% provide 11 or more hours of weekly care.^3^ Informal care is most often provided by women^4^ and by adults in their 50s and 60s.^5^ Informal care is a significant substitute for formal long-term care, saving governments enormous health and social care costs. In 2015, Carers UK estimated that the economic value of the contribution made by carers in the UK was £132 billion per year, almost double its value in 2001.^6^ However, societal impacts and costs should be considered holistically. For example, it is important to understand the costs to national economic productivity if reduction in health and social care costs is achieved at the expense of compromising the quality or quantity of work of individuals providing informal care. In particular, it is important to understand the extent to which the burden of caregiving is affecting those already subject to inequalities of health and wealth, particularly if the caring burden prevents them attaining their own maximal productivity and pension provision. It would be unsustainable for a society if the older adults who provide informal care experience short-term or long-term negative effects on their own health so that they, in turn, require more formal or informal care.

Therefore, we investigated the prevalence of caregiving and the relationship between caring and maintaining paid work, self-reported health and objective health information in the Health and Employment After Fifty (HEAF) study.

## Methods

### Participants

The HEAF study was set up to follow the health, work and retirement experiences of a large population-based cohort of older adults; the study has been described in detail previously.^7^ In brief, during 2013–14, postal questionnaires were mailed to 39 359 adults born between 1948 and 1962 (age at recruitment 50–64 years) from 24 English general practices contributing data to a primary care research database, the Clinical Practice Research Datalink (CPRD). The practices were all over England and all deciles of social deprivation. Ethical approval was obtained from the National Health Service (NHS) Research Ethics Committee North West-Liverpool East (ref: 12/NW/0500).

The baseline questionnaire elicited information about: demographic factors including educational attainment; marital status; anthropometry and lifestyle factors (ever and current smoking and alcohol consumption).

### Informal caregiving

Caregiving responsibilities were elicited from: ‘In an average week, roughly how many hours would you spend giving personal care to someone in your home or family?’ Responses were used to identify participants with ‘any’ vs. ‘no’ caring responsibilities. Additionally, in line with other studies, we defined people who were caregiving for 20 h/week or more as having ‘high-intensity’ caring responsibilities.^8^

### Self-reported health

Standardized self-report tools collected information on: current self-reported health; musculoskeletal pain lasting ≥1 month in the past year in the upper limbs, lower limbs and/or neck/spine and depression [assessed by the Centre for Epidemiologic Studies Depression scale (CES-D) with participants scoring ≥16 considered ‘depressed’].

### Primary care diagnoses

The information extracted from consenting HEAF participants’ CPRD records included: number of consultations in the past 12 months; any previous diagnosis of COPD, diabetes mellitus, hypertension or cardiac conditions and a diagnosis of a regional musculoskeletal pain condition and/or diagnosis of or treatment for a common mental health condition in the preceding 12 months.

### Work factors

Participants reported their employment status and current or most recent occupation (used to derive social class using the Standard Occupational Classification 2010). Current workers provided information about: their weekly working hours (≤20 and >20); shift working (often vs. sometimes/rarely/never); how often they lay awake worrying about work (often vs. sometimes/rarely/never) and job satisfaction (dissatisfied vs. not).

### Statistical analyses

Analyses were carried out separately for men and women, and for people currently in paid work as opposed to those not currently working (unemployed or retired). Summary statistics were used to describe caring responsibilities by socio-demographic and lifestyle characteristics. Logistic regression was used to examine socio-demographic and lifestyle characteristics, work-related factors and health variables, as risk factors for caring for ≥20 h/week (vs. no caring), with results expressed as odds ratios (ORs) and 95% confidence intervals (CIs). To identify which confounders to adjust for, we firstly retained variables significantly associated with the outcome in univariate analysis. When models were mutually adjusted for social class and educational qualification, social class was the dominant socio-economic factor associated with intensive caring and was retained in subsequent models. Additionally, BMI and smoking were no longer significant when added to the multivariate model. Therefore, all subsequent analyses were adjusted for age and social class. Missing data were assumed to be missing at random. Analyses were conducted using Stata (version 15.0).

## Results

In total, 39 359 postal questionnaires were sent, yielding 8134 (20.7%) responses including 3698 men and 4436 women (mean age 58.7 years, SD 4.4). In total, 67.7% were in paid employment, 25.5% were ‘retired’, 6.8% ‘unemployed’, with 11.2% of those not working having stopped because of their health. CPRD data were successfully accessed for 7560 (92.9%) participants. A description of participant characteristics by sex and caring responsibilities is provided in Supplementary table S1.

Table 1 describes informal caregiving among HEAF participants by gender, socio-demographic and lifestyle characteristics. In all, 1153 women (26%) and 644 men (17%) were caregiving for ≥1 h per week. Caregiving was most common amongst those aged 55–59 years (19.2% of men and 28.4% of women) providing a median of 4.0 and 6.0 h/week for men and women, respectively. The estimated total amount of care given by HEAF participants was 20 456 h/week.

**Table 1:** Caring, by sex, socio-demographic and lifestyle characteristics

	Men					Women				
		Among those who are caring					Among those who are caring			
	*N* (%) caring	Hours/week, median (IQR)	*N* (%) caring ≥20 h	OR (95% CI) (≥20 vs. no caring)^a^	OR (95% CI) (≥20 vs. no caring)^b^	*N* (%) caring	Hours/week, median (IQR)	*N* (%) caring ≥20 h	OR (95% CI) (≥20 vs. no caring)^a^	OR (95% CI) (≥20 vs. no caring)^b^
Age class										
50–54	152 (16.0)	4.0 (2.0–10.0)	21 (13.8)	Ref	Ref	288 (24.8)	6.0 (3.0–12.0)	44 (15.3)	Ref	Ref
55–59	225 (19.2)	4.0 (2.0–10.0)	40 (17.8)	1.6 (0.9–2.7)	1.5 (0.9–2.7)	398 (28.4)	6.0 (3.0–12.0)	61 (15.3)	1.2 (0.8–1.8)	1.2 (0.8–1.8)
60–64	267 (17.0)	5.0 (2.0–10.0)	32 (12.0)	0.9 (0.5–1.6)	0.9 (0.5–1.6)	467 (25.0)	8.0 (4.0–15.0)	94 (20.1)	1.3 (0.9–1.9)	1.3 (0.9–1.9)
Social class										
Higher managerial	252 (16.4)	4.0 (2.0–8.0)	20 (7.9)	Ref	Ref	449 (26.2)	6.0 (3.0–10.0)	56 (12.5)	Ref	Ref
Intermediate occupations	157 (19.5)	5.0 (2.0–10.0)	24 (15.3)	2.4 (1.3–4.3)	2.2 (1.2–4.1)	395 (27.5)	7.0 (3.0–14.0)	62 (15.7)	1.3 (0.9–1.9)	1.3 (0.9–1.9)
Routine and manual occupations	222 (17.2)	5.0 (2.0–15.0)	46 (20.7)	2.8 (1.6–4.7)	2.6 (1.5–4.4)	301 (24.5)	10.0 (4.0–20.0)	77 (25.6)	1.9 (1.3–2.7)	1.8 (1.3–2.6)
Educational level										
No qualification/school	180 (15.4)	5.0 (2.0–12.0)	34 (18.9)	2.2 (1.2–4.0)		412 (23.6)	8.0 (4.0–15.0)	89 (21.6)	1.6 (1.1–2.2)	
Vocational training certificate	226 (18.9)	5.0 (2.0–12.0)	42 (18.6)	2.8 (1.6–4.9)		356 (28.5)	7.0 (4.0–14.0)	65 (18.3)	1.7 (1.2–2.5)	
University degree/higher	238 (17.9)	4.0 (2.0–8.0)	17 (7.1)	Ref		385 (26.8)	5.0 (2.0–10.0)	45 (11.7)	Ref	
Marital status										
Single/widowed/divorced	156 (16.1)	6.0 (3.0–12.0)	31 (19.9)	Ref		320 (23.0)	7.0 (3.0–14.0)	56 (17.5)	Ref	
Married/civil partnership	487 (17.9)	4.0 (2.0–10.0)	62 (12.7)	0.7 (0.5–1.1)		819 (27.3)	7.0 (3.0–14.0)	142 (17.3)	1.2 (0.9–1.7)	
Smoking										
Never	323 (17.9)	4.0 (2.0–10.0)	35 (10.8)	Ref	Ref	651 (26.1)	7.0 (3.0–12.0)	100 (15.4)	Ref	Ref
Ex/current	318 (17.1)	5.0 (2.0–10.0)	58 (18.2)	1.6 (1.0–2.5)	1.4 (0.9–2.2)	486 (25.7)	7.0 (3.0–14.0)	97 (20.0)	1.3 (0.9–1.7)	1.2 (0.9–1.6)
BMI										
<25 (underweight/normal)	194 (18.5)	4.0 (2.0–10.0)	22 (11.3)	Ref	Ref	473 (26.2)	6.0 (3.0–12.0)	67 (14.2)	Ref	Ref
≥25 (overweight/obese)	440 (17.2)	5.0 (2.0–10.0)	70 (15.9)	1.3 (0.8–2.1)	1.2 (0.7–1.9)	642 (25.8)	8.0 (4.0–14.0)	126 (19.6)	1.4 (1.0–1.8)	1.3 (0.9–1.8)

OR (95% CI), odds ratio and 95% confidence interval; *N* (%), number and percentage; IQR, inter-quartile range; BMI, body mass index.

a Unadjusted.

b Mutually adjusted for age, social class, smoking and BMI.

Table 1 also shows the age- and sex-specific prevalence of those providing high-intensity care (≥20 h/week). In total, 93 men and 199 women were providing high-intensity care with most care provided by men aged 55–59 years (17.8%) and women aged 60–64 years (20.1%). High-intensity carers vs. those not caring were disadvantaged (by social class or educational level) and were more likely obese and ever/current smokers. Mutual adjustment made little difference to the estimated ORs but suggested that social class (routine and manual vs. higher managerial occupations in both men and women and also intermediate occupations in men) was the factor most strongly associated with intensive caregiving responsibilities.

Table 2 describes the sex-specific employment status of those providing high-intensity care. Even after adjustment for age and social class, high-intensity caring vs. not caring was 4–5-fold more likely amongst unemployed people and 2–3-fold more likely among retired respondents. Subsequent analyses considered non-workers and workers separately.

**Table 2:** Risk of high-intensity caring, by employment status and sex

	Men					Women				
Employment status	*N* (%) no caring	*N* (%) caring 1–19 h	*N* (%) caring ≥20 h	OR (95% CI) (≥20 vs. no caring)^a^	OR (95% CI) (≥20 vs. no caring)^b^	*N* (%) no caring	*N* (%) caring 1–19 h	*N* (%) caring ≥20 h	OR (95% CI) (≥20 vs. no caring)^a^	OR (95% CI) (≥20 vs. no caring)^b^
Employed	1733 (83.4)	312 (15.0)	32 (1.5)	Ref	Ref	1862 (75.4)	536 (21.7)	71 (2.9)	Ref	Ref
Self-employed	503 (82.5)	98 (16.1)	9 (1.5)	1.0 (0.5–2.0)	0.9 (0.4–2.0)	257 (71.0)	86 (23.8)	19 (5.3)	1.9 (1.1–3.3)	2.1 (1.2–3.5)
Unemployed	192 (83.5)	16 (7.0)	22 (9.6)	6.2 (3.5–10.9)	5.1 (2.8–9.1)	206 (68.2)	62 (20.5)	34 (11.3)	4.3 (2.8–6.7)	4.0 (2.6–6.3)
Retired	626 (80.2)	125 (16.0)	30 (3.8)	2.6 (1.6–4.3)	3.8 (2.2–6.8)	958 (73.5)	270 (20.7)	75 (5.8)	2.1 (1.5–2.9)	2.1 (1.4–3.2)

OR (95% CI), odds ratio and 95% confidence interval; *N* (%), number and percentage.

a Unadjusted.

b Adjusted for age and social class.

Table 3 summarizes the health of non-working (unemployed/retired) HEAF participants by level of caring responsibilities and gender. Men (*n* = 52) and women (*n* = 109) in the high-intensity caring group compared with those with no caring responsibilities were more likely to have elevated CES-D depression scores (adjusted OR 2.0 and 1.5, respectively). Intense caring was associated with a higher prevalence of sleep problems in men and musculoskeletal pain in women. Case numbers for many specific CPRD diagnoses were low once cross-classified by employment, gender and caring status. However, the prevalence of COPD was notably higher among men with intense caring responsibilities as compared with those without (adjusted OR 3.6, 95% CI 1.5–8.8), and common mental health problems, diabetes and regional pain were also more prevalent. No clear associations were observed among women.

**Table 3:** Health profile of non-workers, by intensity of caring responsibilities

	Men				Women			
	Total	No caring	1–19 h	≥20 h	Total	No caring	1–19 h	≥20 h
Any MS pain, *N* (%)	302 (30.1)	250 (30.8)	34 (24.3)	18 (34.6)	526 (33.0)	386 (33.4)	91 (27.6)	49 (45.0)
Unadjusted OR (95% CI)		Ref	0.7 (0.5–1.1)	1.2 (0.7–2.2)		Ref	0.8 (0.6–1.0)	1.6 (1.1–2.4)
Adjusted for social class and age, OR (95% CI)		Ref	0.8 (0.5–1.2)	0.9 (0.5–1.7)		Ref	0.8 (0.6–1.1)	1.5 (1.0–2.3)
Poor/fair SRH, *N* (%)	368 (37.1)	304 (37.9)	39 (28.5)	25 (48.1)	480 (30.6)	370 (32.4)	72 (22.3)	38 (35.9)
Unadjusted OR (95% CI)		Ref	0.7 (0.4–1.0)	1.5 (0.9–2.7)		Ref	0.6 (0.4–0.8)	1.2 (0.8–1.8)
Adjusted for social class and age, OR (95% CI)		Ref	0.7 (0.5–1.1)	1.3 (0.7–2.3)		Ref	0.6 (0.5–0.9)	1.1 (0.7–1.7)
CESD score ≥16, *N* (%)	274 (27.6)	220 (27.5)	29 (20.6)	25 (49.0)	434 (27.5)	317 (27.8)	77 (23.4)	40 (37.0)
Unadjusted OR (95% CI)		Ref	0.7 (0.4–1.1)	2.5 (1.4–4.5)		Ref	0.8 (0.6–1.1)	1.5 (1.0–2.3)
Adjusted for social class and age, OR (95% CI)		Ref	0.8 (0.5–1.2)	2.0 (1.1–3.8)		Ref	0.9 (0.6–1.2)	1.5 (1.0–2.3)
Sleep problems	200 (19.8)	167 (2.4)	14 (9.9)	19 (36.5)	363 (22.6)	260 (22.3)	78 (23.5)	25 (22.9)
Unadjusted OR (95% CI)		Ref	0.4 (0.2–0.8)	2.2 (1.2–4.0)		Ref	1.1 (0.8–1.4)	1.0 (0.6–1.7)
Adjusted for social class and age, OR (95% CI)		Ref	0.5 (0.3–0.9)	2.0 (1.1–3.8)		Ref	1.1 (0.8–1.5)	1.0 (0.6–1.5)
Health from CPRD								
Consultations in the year before baseline, median(IQR)	6.0 (2.0,12.0)	6.0 (2.0,12.0)	4.0 (1.0–9.0)	8.0 (3.0–14.5)	5.0 (2.0–10.0)	5.0 (2.0–10.0)	4.0 (1.0–9.0)	5.0 (2.0–9.0)
Common mental health diagnosis OR mood dis order prescription—12 months before	181 (19.3)	149 (19.6)	20 (15.4)	12 (25.0)	376 (25.4)	279 (26.1)	70 (22.5)	27 (27.3)
Unadjusted OR (95% CI)		Ref	0.7 (0.4–1.2)	1.4 (0.7–2.7)		Ref	0.8 (0.6–1.1)	1.1 (0.7–1.7)
Adjusted for social class and age, OR (95% CI)		Ref	0.8 (0.5–1.4)	1.2 (0.6–2.5)		Ref	0.9 (0.6–1.2)	0.9 (0.6–1.5)
Hypertension—ever before	316 (33.7)	264 (34.7)	39 (30.0)	13 (27.1)	395 (26.7)	290 (27.1)	77 (24.8)	28 (28.3)
Unadjusted OR (95% CI)		Ref	0.8 (0.5–1.2)	0.7 (0.4–1.3)		Ref	0.9 (0.7–1.2)	1.1 (0.7–1.7)
Adjusted for social class and age, OR (95% CI)		Ref	0.8 (0.5–1.2)	0.7 (0.3–1.3)		Ref	0.9 (0.7–1.2)	1.1 (0.7–1.7)
Diabetes—ever before	151 (16.1)	123 (16.2)	17 (13.1)	11 (22.9)	146 (9.9)	109 (10.2)	31 (10.0)	6 (6.1)
Unadjusted OR (95% CI)		Ref	0.8 (0.5–1.3)	1.5 (0.8–3.1)		Ref	1.0 (0.6–1.5)	0.6 (0.2–1.3)
Adjusted for social class and age, OR (95% CI)		Ref	0.7 (0.4–1.3)	1.4 (0.7–2.9)		Ref	1.1 (0.7–1.6)	0.5 (0.2–1.3)
Diagnosis COPD—ever before	49 (5.2)	36 (4.7)	6 (4.6)	7 (14.6)	57 (3.9)	44 (4.1)	7 (2.3)	6 (6.1)
Unadjusted OR (95% CI)		Ref	1.0 (0.4–2.4)	3.4 (1.4–8.2)		Ref	0.5 (0.2–1.2)	1.5 (0.6–3.6)
Adjusted for social class and age, OR (95% CI)		Ref	1.1 (0.4–2.7)	3.6 (1.5–8.8)		Ref	0.6 (0.3–1.4)	1.6 (0.6–3.8)
Cardiac conditions—ever before	127 (13.5)	109 (14.3)	15 (11.5)	3 (6.3)	107 (7.2)	87 (8.1)	16 (5.1)	4 (4.0)
Unadjusted OR (95% CI)		Ref	0.8 (0.4–1.4)	0.4 (0.1–1.3)		Ref	0.6 (0.4–1.1)	0.5 (0.2–1.3)
Adjusted for social class and age, OR (95% CI)		Ref	0.7 (0.4–1.4)	0.4 (0.1–1.3)		Ref	0.7 (0.4–1.2)	0.5 (0.2–1.4)
Regional pain—12 months before	177 (18.9)	148 (19.5)	17 (13.1)	12 (25.0)	328 (22.2)	237 (22.2)	67 (21.5)	24 (24.2)
Unadjusted OR (95% CI)		Ref	0.6 (0.4–1.1)	1.4 (0.7–2.7)		Ref	1.0 (0.7–1.3)	1.1 (0.7–1.8)
Adjusted for social class and age, OR (95% CI)		Ref	0.6 (0.3–1.0)	1.1 (0.5–2.2)		Ref	1.0 (0.7–1.4)	1.0 (0.6–1.7)

Ref, reference category; MS, musculoskeletal; SRH, self-rated health; CES-D, Centre for Epidemiologic Studies Depression Scale; CPRD, Clinical Practice Research Datalink; COPD, chronic obstructive pulmonary disease; OR (95% CI), odds ratio and 95% confidence interval; *N* (%), number and percentage.

Table 4 describes the employment characteristics and health of working (employed or self-employed) HEAF participants, by level of caring responsibilities and gender (41 men and 90 women with high-intensity caring). Men and women providing high-intensity care were more likely to be working part-time or shifts than non-carers. Women with heavy caring responsibilities were also more likely to lie awake worrying about work.

**Table 4:** Health and work profile of workers, by intensity of caring responsibilities

	Men				Women			
	Total	No caring	1–19 h	≥20 h	Total	No caring	1–19 h	≥20 h
Work characteristics								
PT, *N* (%)	217 (8.1)	165 (7.4)	46 (11.2)	6 (14.6)	602 (21.3)	422 (19.9)	151 (24.3)	29 (32.2)
Unadjusted OR (95% CI)		Ref	1.6 (1.1–2.2)	2.2 (0.9–5.2)		Ref	1.3 (1.0–1.6)	1.9 (1.2–3.0)
Adjusted for social class and age, OR (95% CI)		Ref	1.6 (1.1–2.3)	3.4 (1.4–8.6)		Ref	1.4 (1.1–1.7)	1.7 (1.1–2.8)
Often shift work, *N* (%)	422 (16.2)	339 (15.6)	70 (17.5)	13 (32.5)	421 (15.1)	312 (15.0)	90 (14.7)	19 (22.1)
Unadjusted OR (95% CI)		Ref	1.2 (0.9–1.5)	2.6 (1.3–5.1)		Ref	1.0 (0.8–1.3)	1.6 (1.0–2.7)
Adjusted for social class and age, OR (95% CI)		Ref	1.2 (0.9–1.6)	1.9 (1.0–3.9)		Ref	1.0 (0.8–1.3)	1.6 (0.9–2.7)
Often lying awake worrying about work, *N* (%)	288 (10.9)	228 (10.4)	54 (13.5)	6 (15.0)	417 (14.9)	281 (13.4)	112 (18.1)	24 (27.3)
Unadjusted OR (95% CI)		Ref	1.3 (1.0–1.8)	1.5 (0.6–3.7)		Ref	1.4 (1.1–1.8)	2.4 (1.5–3.9)
Adjusted for social class and age, OR (95% CI)		Ref	1.3 (1.0–1.8)	1.5 (0.6–3.7)		Ref	1.4 (1.1–1.8)	2.9 (1.8–4.8)
Job dissatisfaction, *N* (%)	198 (7.5)	159 (7.2)	36 (9.0)	3 (7.7)	170 (6.1)	121 (5.8)	44 (7.1)	5 (5.7)
Unadjusted OR (95% CI)		Ref	1.3 (0.9–1.8)	1.1 (0.3–3.5)		Ref	1.3 (0.9–1.8)	1.0 (0.4–2.5)
Adjusted for social class and age, OR (95% CI)		Ref	1.3 (0.9–1.9)	0.9 (0.3–2.9)		Ref	1.2 (0.9–1.8)	1.0 (0.4–2.6)
Health status								
Any MS pain, *N* (%)	605 (22.7)	492 (22.2)	101 (24.9)	12 (29.3)	709 (25.2)	511 (24.3)	161 (25.9)	37 (42.1)
Unadjusted OR (95% CI)		Ref	1.2 (0.9–1.5)	1.4 (0.7–2.9)		Ref	1.1 (0.9–1.3)	2.3 (1.5–3.5)
Adjusted for social class and age, OR (95% CI)		Ref	1.2 (0.9–1.5)	1.3 (0.6–2.5)		Ref	1.1 (0.9–1.4)	2.0 (1.3–3.2)
Poor/fair SRH, *N* (%)	489 (18.4)	397 (18.0)	83 (20.3)	9 (22.0)	497 (17.9)	355 (17.1)	115 (18.7)	27 (30.7)
Unadjusted OR (95% CI)		Ref	1.2 (0.9–1.5)	1.3 (0.6–2.7)		Ref	1.1 (0.9–1.4)	2.1 (1.3–3.4)
Adjusted for social class and age, OR (95% CI)		Ref	1.2 (0.9–1.5)	1.2 (0.6–2.5)		Ref	1.1 (0.9–1.4)	1.9 (1.2–3.0)
CESD score ≥16, *N* (%)	538 (20.2)	430 (19.4)	95 (23.3)	13 (32.5)	774 (27.5)	556 (26.4)	177 (28.7)	41 (46.1)
Unadjusted OR (95% CI)		Ref	1.3 (1.0–1.6)	2.0 (1.0–3.9)		Ref	1.1 (0.9–1.4)	2.4 (1.6–3.7)
Adjusted for social class and age, OR (95% CI)		Ref	1.3 (1.0–1.6)	1.8 (0.9–3.6)		Ref	1.1 (0.9–1.4)	2.2 (1.4–3.4)
Sleep problems, *N* (%)	344 (12.8)	271 (12.1)	65 (15.9)	8 (19.5)	609 (21.5)	437 (20.6)	137 (22.0)	35 (38.9)
Unadjusted OR (95% CI)		Ref	1.4 (1.0–1.8)	1.8 (0.8–3.8)		Ref	1.1 (0.9–1.4)	2.4 (1.6–3.8)
Adjusted for social class and age, OR (95% CI)		Ref	1.3 (1.0–1.8)	1.5 (0.7–3.3)		Ref	1.1 (0.9–1.4)	2.3 (1.5–3.6)
Health from CPRD								
Consultations in the year be fore baseline, median(IQR)	4.0 (1.0,8.0)	4.0 (1.0,8.0)	3.0 (1.0–7.0)	2.5 (1.0–5.0)	3.0 (1.0–7.0)	3.0 (1.0–7.0)	3.5 (1.0–7.0)	5.0 (2.5–9.0)
Common mental health diag nosis OR mood disorder prescription—12 month before	248 (9.9)	203 (9.7)	41 (10.8)	4 (10.5)	545 (20.8)	402 (20.6)	114 (19.5)	29 (34.5)
Unadjusted OR (95% CI)		Ref	1.1 (0.8–1.6)	1.1 (0.4–3.1)		Ref	0.9 (0.7–1.2)	2.0 (1.3–3.2)
Adjusted for social class and age, OR (95% CI)		Ref	1.2 (0.8–1.7)	1.1 (0.4–3.1)		Ref	0.9 (0.7–1.2)	2.0 (1.2–3.1)
Hypertension—ever before	600 (23.8)	504 (24.0)	88 (23.1)	8 (21.1)	438 (16.7)	332 (17.0)	90 (15.4)	16 (19.1)
Unadjusted OR (95% CI)		Ref	1.0 (0.7–1.2)	0.8 (0.4–1.9)		Ref	0.9 (0.7–1.1)	1.2 (0.7–2.0)
Adjusted for social class and age, OR (95% CI)		Ref	0.9 (0.7–1.2)	1.0 (0.4–2.2)		Ref	0.9 (0.7–1.1)	1.1 (0.6–1.9)
Diabetes—ever before	254 (10.1)	214 (10.2)	38 (10.0)	2 (5.3)	161 (6.1)	130 (6.7)	26 (4.4)	5 (6.0)
Unadjusted OR (95% CI)		Ref	1.0 (0.7–1.4)	0.5 (0.1–2.0)		Ref	0.7 (0.4–1.0)	0.9 (0.4–2.2)
Adjusted for social class and age, OR (95% CI)		Ref	1.0 (0.7–1.4)	0.5 (0.1–2.2)		Ref	0.6 (0.4–1.0)	0.9 (0.3–2.2)
Diagnosis COPD—ever before	63 (2.5)	47 (2.2)	13 (3.4)	3 (7.9)	44 (1.7)	33 (1.7)	10 (1.7)	1 (1.2)
Unadjusted OR (95% CI)		Ref	1.5 (0.8–2.9)	3.7 (1.1–12.6)		Ref	1.0 (0.5–2.1)	0.7 (0.1–5.2)
Adjusted for social class and age, OR (95% CI)		Ref	1.6 (0.9–3.1)	4.5 (1.3–15.8)		Ref	1.1 (0.5–2.2)	0.6 (0.1–4.8)
Cardiac conditions—ever before	205 (8.1)	172 (8.2)	31 (8.1)	2 (5.3)	63 (2.4)	47 (2.4)	14 (2.4)	2 (2.4)
Unadjusted OR (95% CI)		Ref	1.0 (0.7–1.5)	0.6 (0.1–2.6)		Ref	1.0 (0.5–1.8)	1.0 (0.2–4.1)
Adjusted for social class and age, OR (95% CI)		Ref	1.0 (0.7–1.5)	0.7 (0.2–2.9)		Ref	1.0 (0.5–1.8)	0.9 (0.2–3.9)
Regional pain—12 months before	413 (16.4)	332 (15.8)	78 (20.5)	3 (7.9)	517 (19.7)	385 (19.7)	105 (17.9)	27 (32.1)
Unadjusted OR (95% CI)		Ref	1.4 (1.0–1.8)	0.5 (0.1–1.5)		Ref	0.9 (0.7–1.1)	1.9 (1.2–3.1)
Adjusted for social class and age, OR (95% CI)		Ref	1.4 (1.0–1.8)	0.5 (0.1–1.5)		Ref	0.9 (0.7–1.2)	1.8 (1.1–2.9)

PT, part time; Ref, reference category; MS, musculoskeletal; SRH, self-rated health; CES-D, Centre for Epidemiologic Studies Depression Scale; CPRD, Clinical Practice Research Datalink; COPD, chronic obstructive pulmonary disease; OR (95% CI), odds ratio and 95% confidence interval; *N* (%), number and percentage.

Self-reported musculoskeletal pain, poor/fair self-rated health, depression and sleep problems were markedly more common among working women with intense, as opposed to no, caring responsibilities. A high CES-D depression score was also more common among working men with intense, as opposed to no, caring responsibilities but adjustment for social class and age attenuated this association. Supplementary figure S1 displays the prevalence of self-reported health items by gender, working and carer status. As per table 3, case numbers for most CPRD diagnoses were rather low once cross-classified by gender and caring among workers. However, a diagnosis of regional pain, or a common mental health condition, was much more likely among working women with intense caring responsibilities compared with those without. As with non-workers, the prevalence of COPD was higher amongst those with high-intensity caring responsibilities when compared with those without.

## Discussion

We found that 26% of women and 17% of men aged 50–64 years in the HEAF cohort are providing informal care, while 4.5 and 2.5%, respectively, are providing care for ≥20 h/week. The burden is greatest among those from socio-economically disadvantaged backgrounds (in routine/manual or intermediate occupations). Caregivers were more likely to be unemployed or retired, and amongst those working, were less likely to be working full-time and more likely to work shifts. Caregiving for ≥20 h/week was associated with self-reported morbidity in both men and women, particularly amongst working women who reported poorer general health and more depression, musculoskeletal pain and sleep problems than women without caregiving responsibilities. Analysis of objective health information from primary care records showed that caregiving for ≥20 h/week was associated with a higher prevalence of COPD in men and a higher prevalence of common mental health conditions or regional pain in the past year in working women.

Other UK-based researchers have reported that caregiving for ≥20 h/week impacted employment and that employment status affected willingness to care.^8–10^ In the EXTEND study, the combination of work and caring responsibilities often resulted in unintentional part-time work, involuntary early retirement and financial insecurity.^11^ It is possible that people with caring responsibilities opt for more routine occupations or choose patterns of shift work to enable them to deliver care or alternatively that people with the worst health opt to reduce their work engagement and then, when a family member needs care, have no choice but to provide this themselves. As such, our findings are consistent with a scenario whereby the socio-economically advantaged HEAF participants with higher managerial occupations are in a position to pay for private care for their family members, whereas those in less optimal circumstances have no choice but to deliver the care themselves. An additional explanation may be that social inequalities in health^12^ mean that the family members of socio-economically advantaged HEAF participants are in generally better health, with lower care needs, than the families of socially disadvantaged HEAF participants.

That caregiving has health impacts have been reported before. In particular, a high prevalence of psychological distress, depression and anxiety amongst caregivers has been found in a number of other studies.^13–15^ Physical health impacts have been less frequently studied. Where they have, researchers have either focussed on only one health outcome^16^ or have considered health care use^17^ or physiological measures^18^ as indicators for physical health^19^^,^^20^ or have relied on self-reported diagnoses without objective health information. Studies in Brazil^15^ and Australia,^21^ for example, asked participants to self-report diagnoses ‘made by a physician’ or conditions that ‘a doctor had ever told them they have or were currently receiving treatment or medication for’. Other researchers have collected health information by asking participants to check a list including a number of conditions.^22^ Whilst these studies consistently suggest a higher burden of physical ill-health in caregivers, an earlier meta-analysis of 23 studies found that stronger relationships occurred with elevated levels of stress hormones, attenuation of numbers of antibodies and poorer global reported health than with other objective disease.^20^ For some types of health conditions (e.g. asthma and arthritis), self-reported diagnoses have low validity. Therefore, it is a particular strength of the current study that objective health diagnoses were available. In our study, and in contrast with some others, high-intensity caring was not consistently associated with a diagnosis of hypertension, cardiac disease or diabetes mellitus; this may be partly due to low numbers of these diagnoses once the sample was stratified by gender and employment status. However, we found that a diagnosis of COPD was more common among high-intensity male caregivers, irrespective of employment status. To our knowledge no directly comparable data are available, but Stacey et al. collected self-reported information amalgamating ‘asthma or COPD’ and found an increased prevalence in caregivers.^21^ COPD is a common condition which is largely smoking-related.^23^ It is therefore not surprising that high-intensity caring was also associated with ever/current smoking in men in our unadjusted analyses, a finding consistent with previously published research.^15^^,^^21^ Although this finding requires replication in longitudinal studies, the association with COPD is potentially important as COPD is the fourth leading cause of death worldwide and causes substantial burden, notably on caregivers.^24^

Consistent with previous studies, we found that self-reported ill-health is more common among caregivers, particularly high-intensity caregivers. Poor/fair self-rated health, high CES-D depression scores, pain and sleep disturbance all tended to be more common in both men and women in this group, but especially in those women also working. Comparing self-reported ill-health with the objective diagnoses, we generally found that the associations between caregiving and ill-health were somewhat weaker for the objective diagnoses. However, among working women, high-intensity care-giving was associated with poorer mental health, and with pain, irrespective of the method of ascertainment. It is possible that some of the more contrasting self-reported results arose due to reporting bias if, for example, individuals providing care are more likely to self-report symptoms than non-caregivers, although there is no reason to think that likely and the questions about caring were not prominent in our questionnaire which covered all aspects of health and work. Alternative explanations are possible. First, our results for self-reported health may reflect a high burden of symptoms in caregivers not troublesome enough to seek health care. Second, caregivers may find it difficult to devote time to their own health care, including making appointments or seeking treatment. This hypothesis was investigated in one Australian study^25^ which unfortunately had a low response rate (24%) and relied on recall but found that carers with chronic disease spent more time managing their own health care than non-caregivers with chronic disease but that the carers with chronic disease also spent more time caring for others than on caring for themselves. Our results add to a growing body of evidence that high-intensity caregivers are more likely than non-caregivers to experience troublesome ill-health. We suggest that cumulatively, this will create increasing difficulties in coping with caring and also with remaining in employment. Sleep disturbance for example, has been found in other studies and been shown to be associated with reduced quality of life^26^ and carer fatigue.^27^ The additional fatigue and burden of caregiving may compromise both the health of the caregiver and the safety and wellbeing of the person for whom care is being provided, perhaps risking the loss of their independence and the likelihood of institutionalisation.

Our findings need to be considered alongside the strengths and limitations of our study. First, these are cross-sectional data so causal relationships cannot be inferred and the possibility of selection bias and reverse causality needs to be acknowledged. For example, the strain of caregiving might increase the risk of an unhealthy lifestyle and poorer health behaviours worsening health or, alternatively, people in poorer health may be more likely to restrict their paid employment so that they are more available to take on caring and have not the financial means to pay for care. Second, although the initial sampling frame was community-based and comprehensive, the response at baseline was fairly low, meaning that caution should be used when generalizing these findings. However, we have previously shown that the sample is reasonably representative of UK in terms of employment status, ethnicity and marital status, as well as including participants from most English regions and all deciles of material affluence or deprivation.^7^ Third, HEAF participants self-reported caring responsibilities and estimated the number of hours per week given to caring. Although it can be difficult for people to estimate accurately exactly how many hours they do specific activities each week, it is likely that non-caregivers would be accurate in their responses and that, if any inaccuracy in estimated hours has occurred, it will be amongst those providing some care or high-intensity care. Interestingly, histograms of reported hours of care revealed that the vast majority of caregivers estimated that they gave considerably <10 h/week, and this group were clearly different from those at the other end of the distribution upon whom caring demands amounted to several hours every day (data not shown). Fourth, our questionnaire did not request information about the nature of the care being provided or for whom care was being given (outside the scope of the HEAF study); if there are differential impacts of caregiving depending upon, e.g. how physically demanding it is or emotionally harrowing, then our study design has not allowed any insight into this. These limitations combined with the relatively low overall number of people with intensive caregiving responsibilities mean that our results require replication in other studies.

Finally, the current study benefits from availability of objective health information from CPRD records. We focussed our analyses on diagnostic entities which are clear-cut and have been shown to have good validity, e.g. hypertension, diabetes mellitus and cardiovascular disease. For common mental health conditions and regional musculoskeletal pain, which are very common and can be mild and fluctuating, the analyses were deliberately restricted to consider only cases diagnosed or treated within the past 12 months, to make them as relevant as possible to the current self-reported health and caring demands.

Our findings have important implications for policy makers as they suggest that expecting people to work into their seventh decade will have adverse effects, particularly on those who are most socially disadvantaged. People may either have to drop out of work before pension age to take on caring responsibilities or alternatively they will not be able to care as they will need to stay in work for financial reasons, such that the burden of care will need to be fulfilled by the Government. A third scenario is that people will need to try and both care and work, putting a strain on them and their health so that they in turn will be future needers of care.

## Conclusions 

We have shown that the requirement to be an informal carer is socially patterned and the need to provide intensive caregiving could affect both health and employment. Our research emphasizes the need for increased awareness of the personal consequences of such intensive informal caring and the importance of government level efforts to address the needs of all carers,^28^ and in particular the needs of those who are also in work.

## Supplementary Material

ckaa078_Supplementary_Data

## References

[1] Office for National Statistics. Population Ageing in the United Kingdom Its Constituent Countries and the European Union. Available at: http://www.ons.gov.uk/ons/dcp171776_258607.pdf (19 February 2020, date last accessed).

[2] Pickard L A growing care gap? The supply of unpaid care for older people by their adult children in England to 2032. Ageing Soc 2015;35:96–123.

[3] Verbakel E, Tamlagsronning S, Winstone L, et al Informal care in Europe: findings from the European Social Survey (2014) special module on the social determinants of health. Eur J Public Health 2017;27(Suppl 1):90–5. 10.1093/eurpub/ckw229 28355645

[4] Arber S, Ginn J Gender differences in informal caring. Health Soc Care Commun 2007;3:19–31.

[5] Pickard L Unpaid care and the family In: Smallwood S, Wilson B, editors. Focus on Families. Basingstoke, UK: Palgrave Macmillan, 2007: 19–34.

[6] Buckner L, Yeandle S Valuing Carers 2015: The Rising Value of Carers’ Support, 2015 Available at: http://www.carersuk.org/for-professionals/policy/policy-library/valuing-carers-2015 (19 February 2020, date last accessed).

[7] Palmer KT, Walker-Bone K, Harris EC, et al Health and Employment after Fifty (HEAF): a new prospective cohort study. BMC Public Health 2015;15:1071. 26482655 10.1186/s12889-015-2396-8 PMC4615332

[8] Carmichael F, Charles S, Hulme C Who will care? Employment participation and willingness to supply informal care. J Health Econ 2010;29:182–90. 20004991 10.1016/j.jhealeco.2009.11.003

[9] King D, Pickard L When is a carer’s employment at risk? Longitudinal analysis of unpaid care and employment in midlife in England. Health Soc Care Commun 2013;21:303–14. 10.1111/hsc.12018 23356685

[10] Heitmueller A The chicken or the egg? Endogeneity in labour market participation of informal carers in England. J Health Econ 2007;26:536–59. 17098311 10.1016/j.jhealeco.2006.10.005

[11] Beach B, Bedell G The EXTEND Project: Exploring Pension Reforms, Work and Inequalities. Available at: https://ilcuk.org.uk/the-extend-project-exploring-pension-reforms-work-and-inequalities/ (19 February 2020, date last accessed).

[12] Marmot M Fair Society, Healthy Lives The Marmot Review: Strategic Review of Health Inequalities in England Post-2010. London: The Marmot Review, 2010.

[13] De Fazio P, Ciambrone P, Cerminara G, et al Depressive symptoms in caregivers of patients with dementia: demographic variables and burden. Clin Interv Aging 2015;10:1085–90. 26170648 10.2147/CIA.S74439 PMC4494176

[14] Berglund E, Lytsy P, Westerling R Health and wellbeing in informal caregivers and non-caregivers: a comparative cross-sectional study of the Swedish general population. Health Qual Life Outcomes 2015;13:109. 26216099 10.1186/s12955-015-0309-2 PMC4517403

[15] Laks J, Goren A, Duenas H, et al Caregiving for patients with Alzheimer’s disease or dementia and its association with psychiatric and clinical comorbidities and other health outcomes in Brazil. Int J Geriatr Psychiatry 2016;31:176–85. 26011093 10.1002/gps.4309

[16] Miyawaki A, Tomio J, Kobayashi Y, et al Impact of long hours family caregiving on non-fatal coronary heart disease risk in middle-aged people: results from a longitudinal nationwide study in Japan. Geriatr Gerontol Int 2017;17:2109–15. 28464424 10.1111/ggi.13061

[17] Shaffer KM, Nightingale CL Comparison of healthcare utilization between informal caregivers and non-caregivers: an analysis of the Health Information National Trends Survey. J Aging Health 2019. doi:10.1177/0898264319830262. 10.1177/0898264319830262 PMC7034877 30793639

[18] Mortensen J, Dich N, Clark AJ, et al Informal caregiving and diurnal patterns of salivary cortisol: results from the Whitehall II Cohort Study. Psychoneuro endocrinology 2019;100:41–7. 10.1016/j.psyneuen.2018.09.030 30290284

[19] Pinquart M, Sörensen S Correlates of physical health of informal caregivers: a meta-analysis. J Gerontol B Psychol Sci Soc Sci 2007;62:P126–37. 17379673 10.1093/geronb/62.2.p126

[20] Vitaliano PP, Zhang J, Scanlan JM Is caregiving hazardous to one’s physical health? A meta-analysis. Psychol Bull 2003;129:946–72. 14599289 10.1037/0033-2909.129.6.946

[21] Stacey AF, Gill TK, Price K, Taylor AW Differences in risk factors and chronic conditions between informal (family) carers and non-carers using a population-based cross-sectional survey in South Australia. BMJ Open 2018;8:e020173. 10.1136/bmjopen-2017-020173 PMC6059288 30037861

[22] Sambasivam R, Liu J, Vaingankar JA, et al The hidden patient: chronic physical morbidity, psychological distress and quality of life in caregivers of older adults. Psychogeriatrics 2019;19:65–72. 30182505 10.1111/psyg.12365 PMC6635743

[23] Postma DS, Bush A, van den Berge M Risk factors and early origins of chronic obstructive pulmonary disease. Lancet 2015;385:899–909. 25123778 10.1016/S0140-6736(14)60446-3

[24] Noonan MC, Wingham J, Taylor RS ‘Who Cares?’ The experiences of caregivers of adults living with heart failure, chronic obstructive pulmonary disease and coronary artery disease: a mixed methods systematic review. BMJ Open 2018;8:e020927. 10.1136/bmjopen-2017-020927 PMC6082485 29997137

[25] Jowsey T, McRae I, Gillespie J, et al Time to care? Health of informal older carers and time spent on health related activities: an Australian survey. BMC Public Health 2013;13:374. 23607727 10.1186/1471-2458-13-374 PMC3641944

[26] Lee K, Yiin J, Lin P, Lu S Sleep disturbances and related factors among family caregivers of patients with advanced cancer. Psychooncology 2015;24:1632–8. 25871014 10.1002/pon.3816

[27] Burton LC, Newsom JT, Schulz R, et al Preventive health behaviors among spousal caregivers. Prev Med 1997;26:162–9. 9085384 10.1006/pmed.1996.0129

[28] Department of Health and Social Care. Carers Action Plan 2018 to 2020: Supporting Carers Today, 2018 Available at: https://www.gov.uk/government/publications/carers-action-plan-2018-to-2020 (19 February 2020, date last accessed).

