# Supplementary material for: Relationships between informal caregiving, health and work in the Health and Employment After Fifty study, England
Source: Eur J Public Health. 2020 Jun 3;30(4):799–806. doi: 10.1093/eurpub/ckaa078 (PMC7445037; doi:10.1093/eurpub/ckaa078)
Supplement: ckaa078_Supplementary_Data [file ckaa078_supplementary_data.zip › ejph-2019-06-om-0512-File003.docx]

**Supplementary Table 1: Characteristics of the sample, by sex and caring responsibilities**

|  | **Men, N (%)** | | | | **Women, N (%)** | | | |
| --- | --- | --- | --- | --- | --- | --- | --- | --- |
|  | **total (n=3698)** | **no caring (n=3054)** | **1-19 hrs (n=551)** | **≥20 hrs (n=93)** | **total (n=4436)** | **no caring (n=3283)** | **1-19 hrs (n=954)** | **≥20 hrs (n=199)** |
| Age class |  |  |  |  |  |  |  |  |
| 50-54 | 951 (25.7) | 799 (26.2) | 131 (23.8) | 21 (22.6) | 1161 (26.2) | 873 (26.6) | 244 (25.6) | 44 (22.1) |
| 55-59 | 1174 (31.8) | 949 (31.1) | 185 (33.6) | 40 (43.0) | 1404 (31.7) | 1006 (30.6) | 337 (35.3) | 61 (30.7) |
| 60-64 | 1573 (42.5) | 1306 (42.8) | 235 (42.7) | 32 (34.4) | 1871 (42.2) | 1404 (42.8) | 373 (39.1) | 94 (47.2) |
|  |  |  |  |  |  |  |  |  |
| Social class |  |  |  |  |  |  |  |  |
| Higher managerial | 1540 (42.4) | 1288 (42.9) | 232 (42.9) | 20 (22.2) | 1713 (39.1) | 1264 (39.1) | 393 (41.4) | 56 (28.7) |
| Intermediate occupations | 805 (22.2) | 648 (21.6) | 133 (24.6) | 24 (26.7) | 1439 (32.8) | 1044 (32.3) | 333 (35.1) | 62 (31.8) |
| Routine and manual occupations | 1290 (35.5) | 1068 (35.5) | 176 (32.5) | 46 (51.1) | 1230 (28.1) | 929 (28.7) | 224 (23.6) | 77 (39.5) |
|  |  |  |  |  |  |  |  |  |
| Educational level |  |  |  |  |  |  |  |  |
| No qualification/School | 1168 (31.6) | 988 (32.4) | 146 (26.5) | 34 (36.6) | 1749 (39.4) | 1337 (40.7) | 323 (33.9) | 89 (44.7) |
| Vocational training certificate | 1199 (32.4) | 973 (31.9) | 184 (33.4) | 42 (45.2) | 1250 (28.2) | 894 (27.2) | 291 (30.5) | 65 (32.7) |
| University degree/higher | 1331 (36.0) | 1093 (35.8) | 221 (40.1) | 17 (18.3) | 1437 (32.4) | 1052 (32.0) | 340 (35.6) | 45 (22.6) |
|  |  |  |  |  |  |  |  |  |
| Marital status |  |  |  |  |  |  |  |  |
| single/widowed/divorced | 968 (26.2) | 812 (26.7) | 125 (22.7) | 31 (33.3) | 1392 (31.7) | 1072 (32.9) | 264 (28.1) | 56 (28.3) |
| married/civil partnership | 2721 (73.8) | 2234 (73.3) | 425 (77.3) | 62 (66.7) | 3002 (68.3) | 2183 (67.1) | 677 (71.9) | 142 (71.7) |
|  |  |  |  |  |  |  |  |  |
| Smoking |  |  |  |  |  |  |  |  |
| Never | 1808 (49.4) | 1485 (49.1) | 288 (52.6) | 35 (37.6) | 2491 (56.9) | 1840 (56.7) | 551 (58.6) | 100 (50.8) |
| Ex/current | 1855 (50.6) | 1537 (50.9) | 260 (47.5) | 58 (62.4) | 1891 (43.2) | 1405 (43.3) | 389 (41.4) | 97 (49.2) |
|  |  |  |  |  |  |  |  |  |
| BMI |  |  |  |  |  |  |  |  |
| <25 (Underweight/Normal) | 1048 (29.1) | 854 (28.8) | 172 (31.7) | 22 (23.9) | 1806 (42.0) | 1333 (41.9) | 406 (44.0) | 67 (34.7) |
| ≥ 25 (Overweight/Obese) | 2556 (70.9) | 2116 (71.3) | 370 (68.3) | 70 (76.1) | 2491 (58.0) | 1849 (58.1) | 516 (56.0) | 126 (65.3) |
|  |  |  |  |  |  |  |  |  |
| Employment status |  |  |  |  |  |  |  |  |
| Employed | 2077 (56.2) | 1733 (56.8) | 312 (56.6) | 32 (34.4) | 2469 (55.7) | 1862 (56.7) | 536 (56.2) | 71 (35.7) |
| Self-employed | 610 (16.5) | 503 (16.5) | 98 (17.8) | 9 (9.7) | 362 (8.2) | 257 (7.8) | 86 (9.0) | 19 (9.6) |
| Unemployed | 230 (6.2) | 192 (6.3) | 16 (2.9) | 22 (23.7) | 302 (6.8) | 206 (6.3) | 62 (6.5) | 34 (17.1) |
| Retired | 781 (21.1) | 626 (20.5) | 125 (22.7) | 30 (32.3) | 1303 (29.4) | 958 (29.2) | 270 (28.3) | 75 (37.7) |

The total number of missing values for the above variables ranges between 0 (age, education) and 94 (men) and 139 (women) for BMI
