# Supplementary figures and images for: Relationships between informal caregiving, health and work in the Health and Employment After Fifty study, England
Source: Eur J Public Health. 2020 Jun 3;30(4):799–806. doi: 10.1093/eurpub/ckaa078 (PMC7445037; doi:10.1093/eurpub/ckaa078)

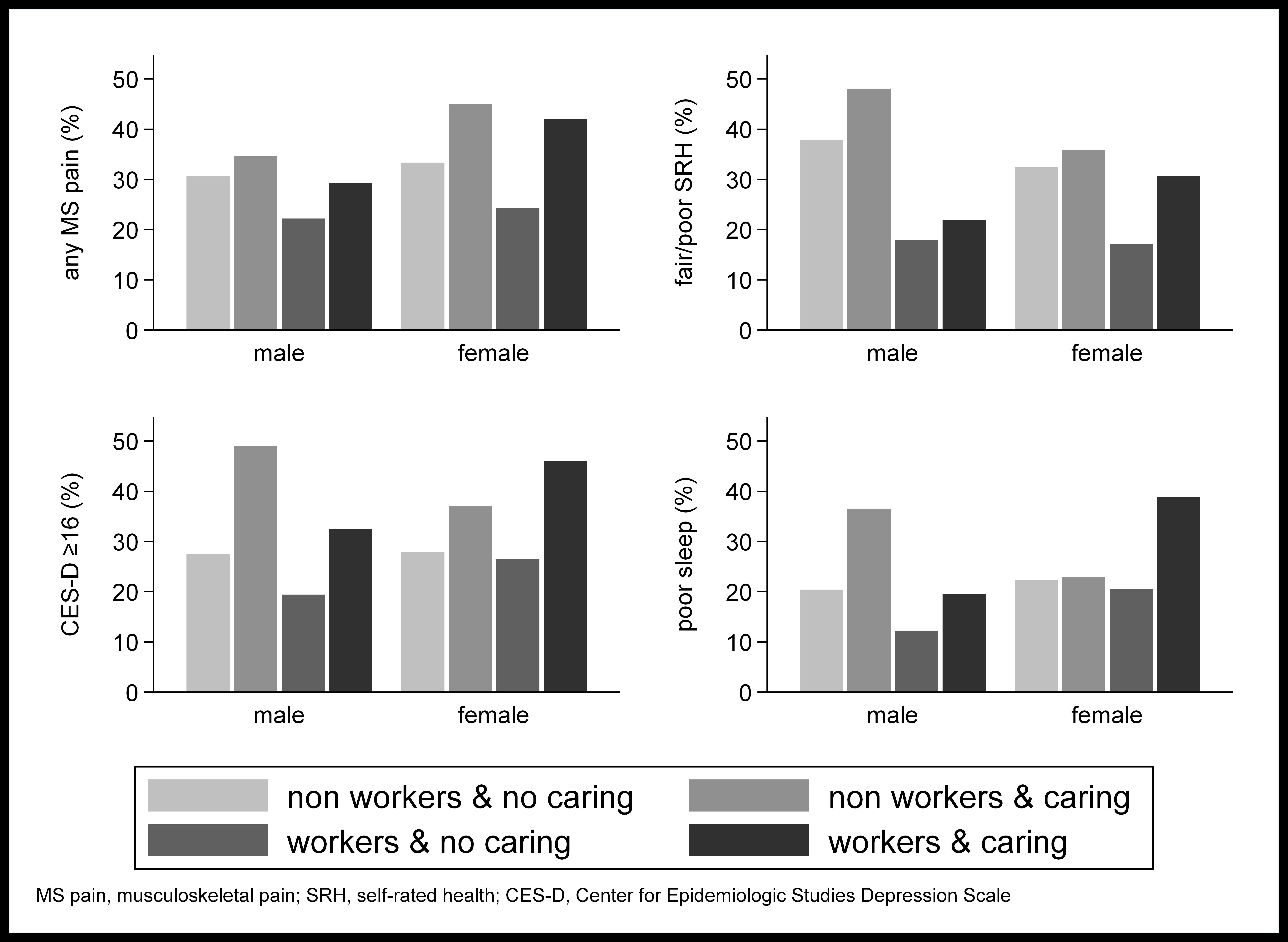

Supplement: ckaa078_Supplementary_Data [file ckaa078_supplementary_data.zip › ejph-2019-06-om-0512-File002.jpg]
